# Plant In Vitro Cultures of *Coleus scutellarioides* (L.) Benth. “Electric Lime” and Possibilities of Modification in the Biosynthesis of Volatile Compounds

**DOI:** 10.3390/molecules29102193

**Published:** 2024-05-08

**Authors:** Maciej Jakobina, Jacek Łyczko, Antoni Szumny, Renata Galek

**Affiliations:** 1 Department of Plant Breeding and Seed Production, Wrocław University of Environmental and Life Sciences, Grunwaldzki Square 24a, 50-363 Wrocław, Poland; renata.galek@upwr.edu.pl; 2 Department of Food Chemistry and Biocatalysis, Wrocław University of Environmental and Life Sciences, Norwida 25, 53-375 Wrocław, Poland; jacek.lyczko@upwr.edu.pl (J.Ł.); antoni.szumny@upwr.edu.pl (A.S.)

**Keywords:** coleus, modification biosynthesis, chemical compounds, phytohormones, in vitro cultures

## Abstract

*Coleus scutellarioides* (L.) Benth. is a globally spread species, known for its characteristic spectacularly colorful leaves of decorative value. Thanks to its rich chemical composition, the plant is used in ethnopharmacology, and it is also regarded as having high medicinal potential. The application of in vitro cultures enables the acquisition of homogeneous certified material of high quality. Additionally, excluding the effect of biotic and abiotic factors on the plants is a way to fully recognize the influence of phytohormones on the plant morphology and the biosynthetic pathways of compound production. The best way to grow *C. scutellarioides* “Electric Lime” under in vitro conditions is to use the basic MS medium (Murashige and Skoog medium), enriched with naphthyl-1-acetic acid at a concentration of 0.5 mg dm^−3^. The analysis of volatile compounds demonstrated that the content of volatile compounds in the plants cultivated under in vivo conditions was expressed at a level of 2848.59 µg g^−1^, whereas in the plants bred in vitro without supplementation with phytohormones, the level was 8191.47 µg g^−1^. The highest content was noted for copaene, α-pinene, 1-octene-3-ol, α-selinene, sabinen, γ- and δ-cadinene, 3-octanol, and β-pinene. Aroma profiling revealed a lack of boranyl acetate, 2-hexenal, and 2-hexen-1-ol in the plants cultivated under in vivo conditions. Differences were found in the volatile composition between plants bred in vivo and in vitro, with the most significant recorded for the contents of 1-octen-3-ol and 3-octanol. The addition of plant growth regulators into the basic medium under in vitro conditions affected the percentage ratio and contents of specific compounds in plant tissues. The most intense biosynthesis of volatile compounds took place in the plants cultivated on the medium enriched with NAA at 10,579.11 µg g^−1^, whereas the least intense was noted for plants cultivated on the medium supplemented with BA, where it was recorded at the level of 5610.02 µg g^−1^. So far, there has been no research published which would pertain to the profiling of volatile compounds performed using the SPME (solid-phase microextraction) technique. Moreover, the very few studies conducted on the chemical composition of these compounds do not mention the specific variety of *C. scutellarioides* under analysis.

## 1. Introduction

The coleus *Coleus scutellarioides* (L.) Benth. belongs to the genus *Coleus* within the mint family *Lamiaceae* Lindl. Based on research carried out in 2018–2019, the genus *Coleus* was created, which includes a few species formerly ranked with the genus *Plectranthus* L’Hér., the species *C. scutellarioides* being identified, among others [1,2].

The generic name coleus comes from the Greek word *koleos*, meaning a shield, and refers to the way the stamens are merged with one another. The epithet *scutellarioides* means “resembling the genus *Scutellaria*”, the name which has been derived from the Latin word *scutella*, denoting a dish or little bowl, with reference to the shape of the calyx, which remains stable after the flowers have withered. Moreover, the term “coleus” is commonly used by gardeners with reference to this particular species [3,4,5].

Investigations show that the tissues of coleus contain rosmarinic acid, which is one of the main components in the plant [6]. The species composition also includes sterols, triterpenoids, campesterol, alfa-amyrine, beta-amyrine, flavonoids, and substances similar to salvinorin of unknown chemical structures, as well as diterpenoids of the abietan type, spirocutelones A-C, coleanol, forskoline, secoabietan, barbutazine, napthopiron, plectryne, crocetine, plectrinon A, deoxycoleonol, acetoxycoleosol, coleol, coleonon, coleon O and G, lanugon K, and 6-acetylfredericon B. Its leaves contain alkaloids, saponines, tannins, quercetin, phenolic compounds, and anthocyanins [7,8,9,10,11]. In raw leaf extract, the occurrence of terpenoids, phenolic compounds, polyphenols, flavonoids, quinones, saponins, and anthraquinones was recorded [12,13]. The content of essential oil in the leaves amounted to 0.04%. The oil is brownish yellow, with a biscuit flavor and a tea fragrance. The main components of the oil are: phytol (21.1%), germacrene-D (9.30%), caryophyllene (8.84%), ß-elemen (6.84%), and neophytadiene (6.08%) [14]. Mass spectrum (GC-MS) examination revealed the largest share of spathulenol (24.57%), germacrene-D (14.53%), bicyclogermacrene (11.24%), nonacosan (9.74%) and morillol (4.85%) [15].

As a medicinal plant, *C. scutellarioides* has been used in folk phytotherapy and has attracted scientific interest due to its properties. The currently pursued research on the species reveals its potential activity as an inhibitor of the histamine H4 receptor [10]. Furthermore, it improves wound healing, inhibits inflammatory and exudative processes, and reduces pain [13]. It also acts against certain cancer cell lines, and exhibits antioxidant, fungistatic, antidiabetic, antipyretic, and immunostimulant properties, as well as antimicrobial activity against *Bacillus subtilis*, *Pseudomonas aeruginosa*, and *Candida albicans* [8,9,16,17,18,19,20].

In the Philippine folk medicine, *C. scutellarioides* leaves are used for the treatment of headaches, maldigestion, sinusitis, bruise healing, and to stop the bleeding from wounds. Advantageous effects have also been recorded for the extract, which is administered in the form of drops, to relieve conjunctivitis. As a traditional Asian plant, the herb is applied in the treatment of angina pectoris, asthma, bronchitis, gastric problems, epilepsy, elephantiasis, insomnia, skin rashes, and scorpion bites. In Southeast Asia, the plant is dedicated to the treatment of dysentery and various gastric problems. In Indonesia, certain varieties are used to treat abscesses, ulcers, and inflammation of the eyes and ears. Roots find application in the treatment of diarrhea and stomach aches, as well as in the therapy of diabetes, constipations, fever, and painful menstruation. On the Vanuatu islands, its extracts are used for abortion, as a contraceptive, or as a remedy for amenorrhea. The leaves are utilized as an emmenagogic agent, but also for improving lactation through the heating and softening of the plant in hot water and applying the preparation onto the nipples [19,21,22,23,24,25].

Thus far, no research has been published focusing on the profiling of volatile compounds using the SPME (solid-phase microextraction) technique. Moreover, in the very few studies on the chemical composition of coleus, there is no mention of the specific varieties of *C. scutellarioides* analyzed in this paper.

The application of plant tissue culture is a very good and fast means to acquire homogeneous plant material in large quantities. Through micropropagation, it is possible to produce numerous clones of a given plant. Material obtained in in vitro culture is characterized by homogeneity, high quality, and repeatability. Employment of this technique in the propagation of medicinal plants allows for a standardized raw material to be achieved. It ensures that plants of documented quality can quickly be introduced into cultivation [26].

The purpose of our study was to produce an in vitro culture of *C. scutellarioides* in order to obtain uniform testing material. The use of growth regulators allowed us to study their effects on the plant under study. Morphological changes in the plant were determined, as well as differences in volatile compounds.

## 2. Results

### 2.1. Plant In Vitro Cultures

The effective induction of cultures was conducted using exclusively stems with nodes (plants without roots/shoots or leaves). The stems themselves showed vitality and produced roots. However, shoot production was not observed.

**Figure 1:**
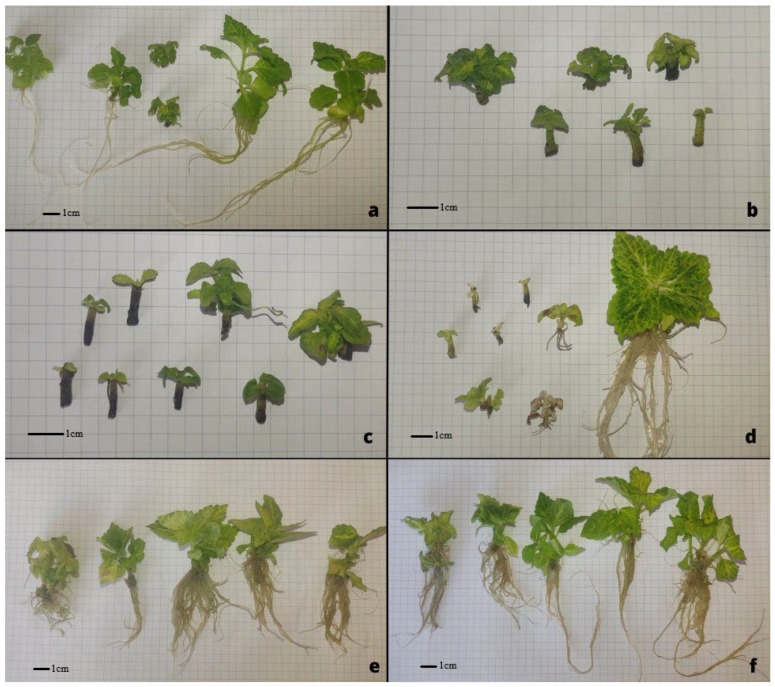
*C. scutellarioides* “Electric Lime” cultivated in vitro under different conditions. (**a**)—MS control medium; (**b**)—MS + 0.5 mg dm^−3^ BA; (**c**)—MS + 0.5 mg dm^−3^ 2iP; (**d**)—MS + 0.5 mg dm^−3^ fluridone; (**e**)—MS + 0.5 mg dm^−3^ NAA; (**f**)—MS + 0.5 mg dm^−3^ IAA. M. Jakobina.

The plants cultivated under in vitro conditions responded differently to the composition of the breeding medium (Figure 1a–f). The stems of plants bred on media supplemented with 0.5 mg dm^−3^ of BA and 0.5 mg dm^−3^ of 2iP turned black at the place of immediate contact with the substratum (Figure 1b,c). Additionally, the root growth cones in the plants cultivated on the medium enriched with 0.5 mg dm^−3^ of fluridone were observed to turn brown, or they assumed the white color (Figure 1d).

The best rooting (96.7% on average) was recorded for the medium supplemented with auxins. Under control conditions, the rooting process was lower by over 8.8% than that observed for the medium enriched with auxin IAA. Cytokinins added to the breeding medium reduced the rooting of explants by 67.7%, whereas the addition of fluridone caused a decline in explants rooting by 19.5%, as compared with the control. The shoot production was found to be the same under each circumstances—all the plants produced shoots. The highest survivability, at a level of 95.8%, was characteristic of those explants which were cultivated on the medium supplemented with auxins. Cytokinins caused the lowering of survivability by over 11.6% in comparison with the control conditions, while fluridone had the worst effect on survivability, reducing it by over 19.5% compared with that of the control.

**Figure 2:**
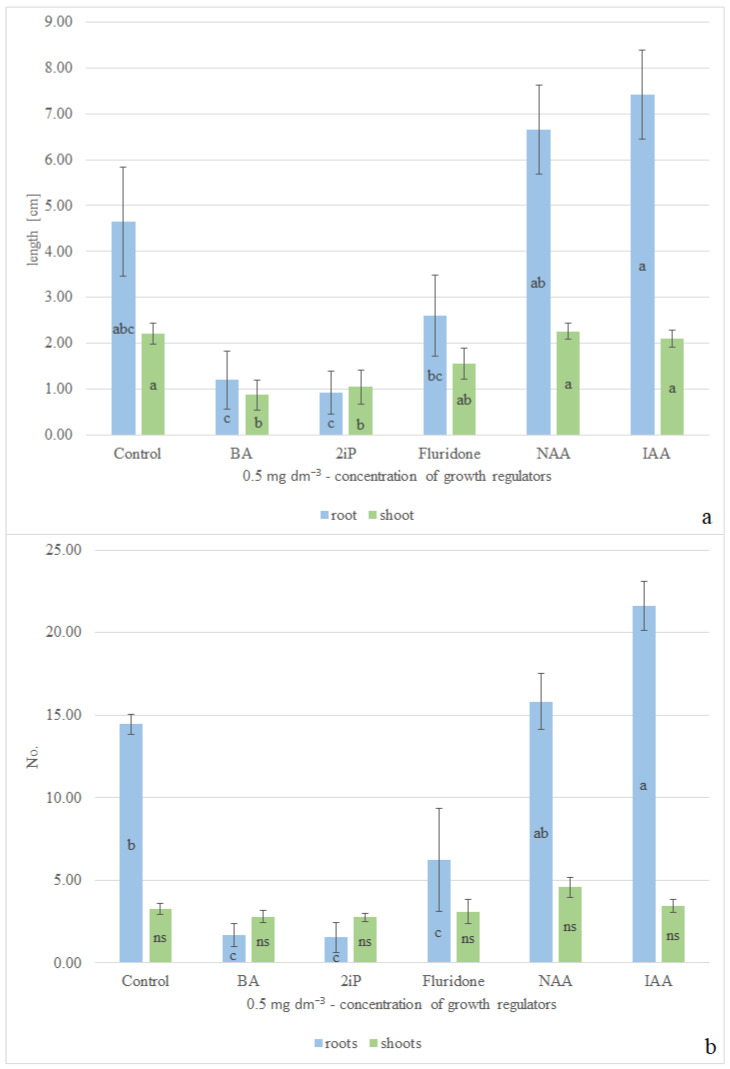
Influence of cultivation conditions on the growth of *C. scutellarioides* “Electric Lime” explants (**a**) influence of supplementation with plant growth regulators on the root length and shoot length; (**b**) influence of addition of phytohormones on the number of roots and shoots. Values followed by a different letter are significantly different (*p* > 0.05, according to the Tukey test). ns—not significant.

The variance analysis performed has shown that the type of phytohormones used affects all of the analyzed traits, except for the number of shoots. The largest mean root length—7.04 cm—was attained on the medium with an addition of IAA and on that enriched with NAA. Supplementation with fluridone resulted in a decrease in the root length by 2 cm compared to plants cultivated on the control medium. The roots were over three times shorter on the breeding medium with BA, and over five times shorter on the medium supplemented with 2iP in comparison with the plants cultivated under control conditions, i.e., without plant growth regulators. The best growth of shoots was observed in the plants bred on the medium enriched with auxins (IAA or NAA) and those bred on the control medium, with shoots over 2 cm long. Fluridone added to the breeding medium caused a decrease in the shoot length by 1.4 times as compared with that of the plants cultivated on the medium lacking phytohormones, whereas the addition of cytokinins (BA or 2iP) resulted in a double reduction of the shoot length as compared with that of the control sample (Figure 2a). The highest mean number of roots was attained in the case of explants bred on the medium enriched with IAA, with a ca 1.5 times more than that noted on the control medium. More than nine times fewer roots were produced on the media supplemented with cytokinins in comparison with the rate under control conditions. The average number of roots on the medium enriched with fluridone equaled 6.22. For the number of shoots—the level of which was 2.75–4.57 cm (Figure 2b)—no statistically significant differences were noted.

### 2.2. Volatile Compounds Composition

Analysis of volatile compounds in *C. scutellarioides*. “Electric Lime” revealed the occurrence of 30 compounds when the plant was cultivated under in vivo conditions, whereas 33 volatile compounds were identified in the plants derived from in vitro cultures (Table 1 and Table 2). The main volatile compounds recognized were: copaene, α-pinene, 1-octen-3-ol, α-selinene, sabinen, γ- and δ-cadinene, 3-octanol, and β-pinene.

**Figure 3:**
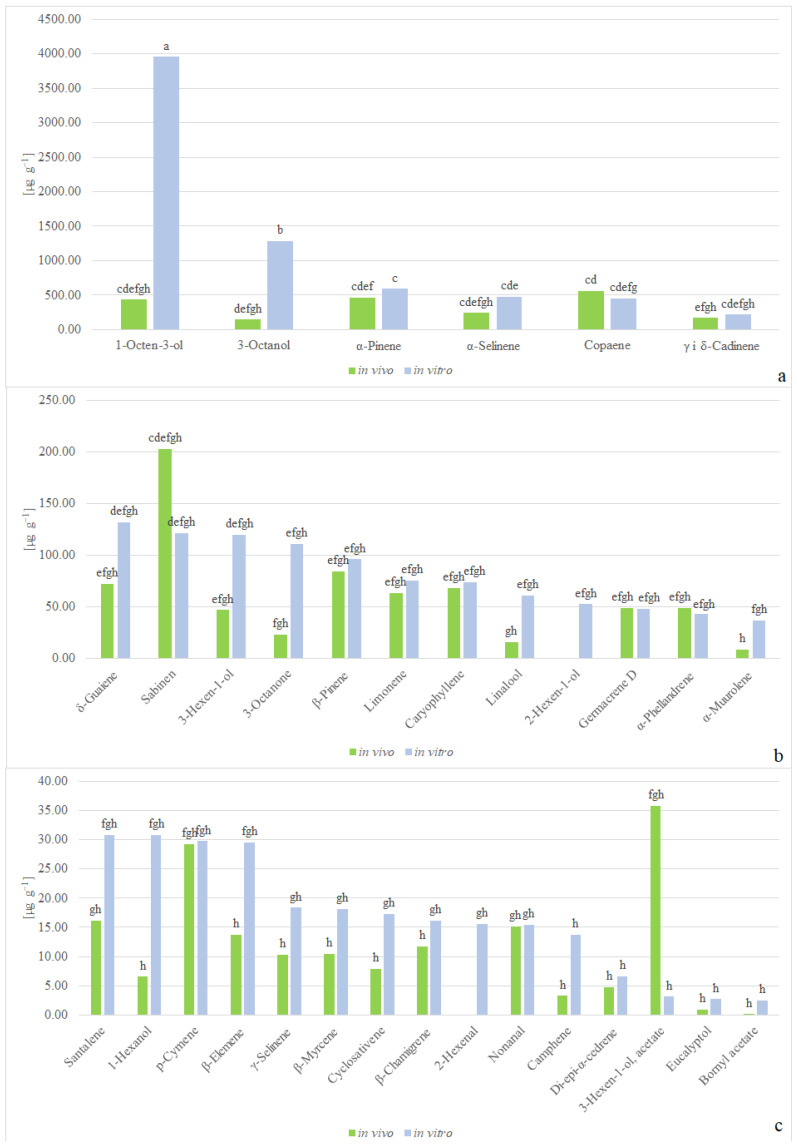
Contents of volatile compounds in *C. scutellarioides* “Electric Lime” tissues: (**a**–**c**) contents of volatile compounds in plants cultivated under in vivo and in vitro conditions, without plant growth regulators. Values followed by a different letter are significantly different (*p* > 0.05, according to the Tukey test).

One-factor variance analysis demonstrated that the content of volatile compounds in the plants cultivated under in vivo conditions was at a level of 2848.59 µg g^−1^, whereas in the plants bred in vitro without supplementation with phytohormones, the content was at a level of 8191.47 µg g^−1^. Moreover, aroma profiling revealed a lack of 2-hexenal and 2-hexen-1-ol, and only trace amounts of boranyl acetate in the plants grown in vivo. The quantities of particular volatile compounds calculated in relation to the internal standards differed between plants grown under in vivo conditions and those bred in in vitro culture. In the plants cultivated in vivo, there was 1.6 times more sabinen and 11.3 times more 3-hexen-1-ol acetate, while under in vitro control conditions, there was 9.1 times more 1-octen-3-ol, 8.8 times more 3-octanol, 4.7 times more 3-octanone, 3.8 times more linalool, and 4.3 times more α-muurolene (Figure 3a–c). The average amount of the compounds reached the level of 10,579.11 µg g^−1^.

The most intense biosynthesis of volatile compounds took place in the plants cultivated on the medium enriched with NAA, whereas the least intense was observed on the medium supplemented with BA, where it was recorded at the level of 5610.02 µg g^−1^. Irrespective of the plant growth regulator added to the breeding medium, supplementation was found to reduce the biosynthesis of 2-hexen-1-ol, α-phellandrene, p-cymene, and nonanal. On the other hand, the biosynthesis of camphene, β-pinene, boranyl acetate, β-myrcene, and eucalyptol was observed to increase, irrespective of the phytohormone applied to the breeding medium. BA caused the highest intensification in the biosynthesis of 3-hexen1-ol acetate, by 4.5 times. Of the employed plant growth regulators, it was only BA which caused a decline in the biosynthesis of linalool, β-elemene, δ-guaiene, α-selinene, and γ-selinene.

Among the phytohormones used, fluridone most significantly enhanced the biosynthesis of α-muurolene (1.2 times), α-pinene (1.3 times), limonene (1.4 times), β-pinene (1.5 times), santalene (2.1 times), linalool and cyclosativene (1.6 times), δ-guaiene (1.9 times), sabinen (3.0 times), and β-chamigrene (7.9 times). On the other hand, fluridone was responsible for the largest drop in the biosynthesis of 1-hexanol and di-*epi*-α-cedrene. As the only one of the applied plant growth regulators, NAA supplemented in the breeding medium improved the biosynthesis of 1-octen-3-ol, 3-octanol, 3-hexen-1-ol, 2-hexanal, and 1-hexanol. This phytohormone was found to most significantly stimulate the biosynthesis of 2-hexenal (1.1 times), 3-hexen-1-ol and γ- and δ-cadinene (1.2 times), 1-octen-3-ol, 3-octanol and 1-hexanol (1.3 times), copaene (1.5 times), α-selinene (1.7 times), β-elemene (2.0 times), and eucalyptol (8.6 times). IAA was the only plant growth regulator supplemented in the breeding medium that was recorded to have a negative impact on the biosynthesis of α-pinene. However, this phytohormone was found to be the best stimulating agent for the biosynthesis of 3-octanone (1.4 times), germacrene-D (1.8 times), caryophyllene (2.3 times), di-*epi*-α-cedrene (3.3 times), camphene (9.9 times), and boranyl acetate (62 times).

**Figure 4:**
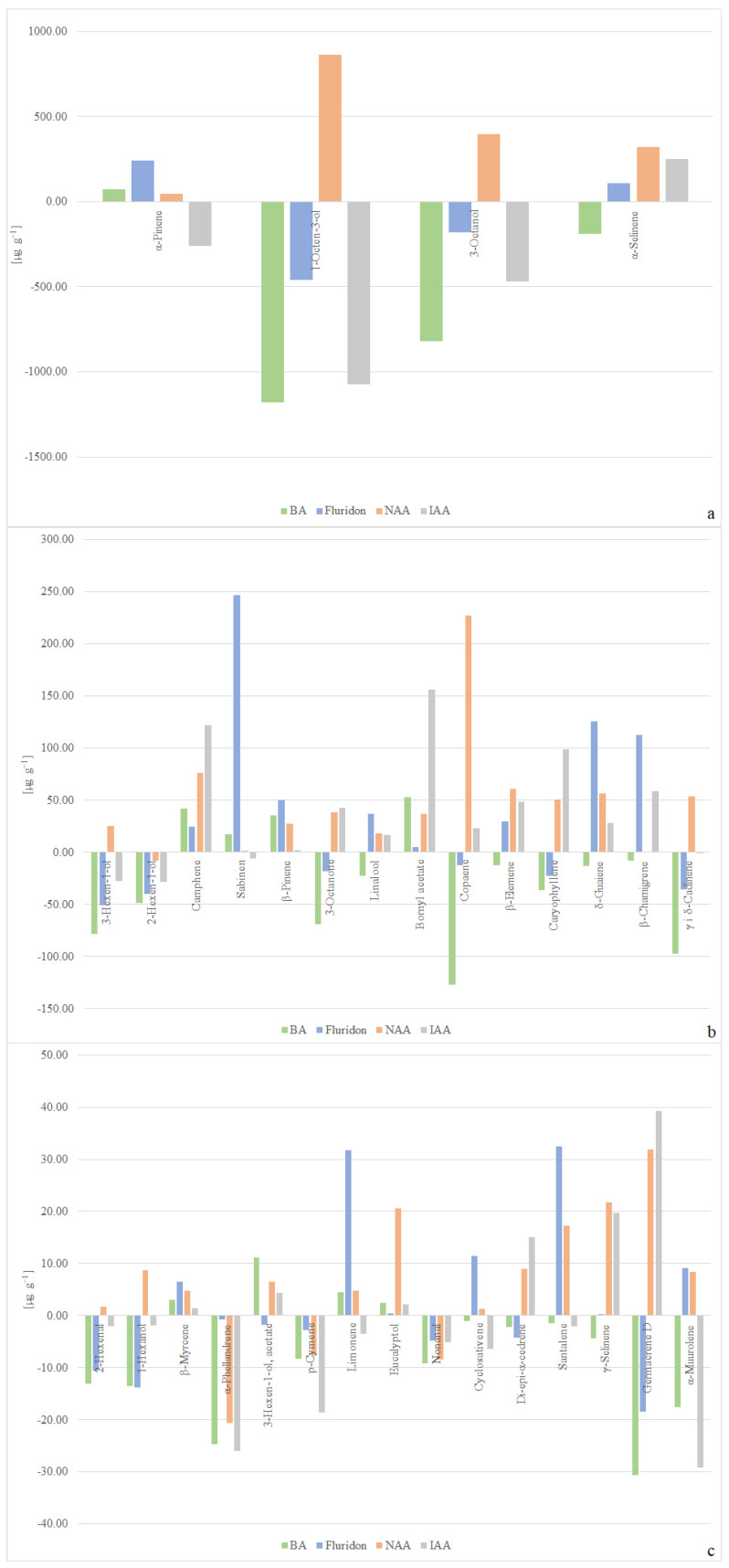
Contents of volatile compounds in *C. scutellarioides* “Electric Lime” tissues: (**a**–**c**) contents of volatile compounds in plants cultivated in vitro with an addition of plant growth regulators as compared with those of the control.

The addition of BA to the medium induces a clear decreasing trend in the context of the content of individual volatile compounds; however, in the case of α-pinene, camphene, sabinene, β-pinene, bornyl acetate, β-myrcene, 3-hexen-1-ol, acetate, limonene, and eucalyptol, it stimulates biosynthesis. Fluridone and IAA stimulated biosynthesis for 17 compounds, while it decreased biosynthesis for 16 compounds. No stimulatory or inhibitory trend is observed. In the case of NAA, a clear stimulating trend is observed for the biosynthesis of volatile compounds, with the exception of 2-hexen-1-ol, α-phellandrene, p-cymene, and nonanal, for which showed a decreasing trend (Figure 4a–c). The calculated correlation coefficients generally confirmed, for most of the analyzed compounds, an increase in their biosynthesis with phytohormone supplementation (Supplementary Table S1).

## 3. Discussion

### 3.1. Discussion of In Vitro Cultivation

The effective induction of aseptic tissue cultures was attained by using stems with nodal segments. Other scientific teams induced tissue culture in *C. scutellarioides* from stems, leaves, or nodal segments comprising lateral buds and from apical shoots [27,28,29,30].

In our study, induction was successful after six weeks. Auxins are reported to positively affect the diversification of cells, elongational growth, and plant rooting. The auxins used in our research, which are NAA (naphthyl-1-acetate acid) and IAA (indolil-3-acetate acid), exerted such a stimulating influence on the studied plants. Although the differences were not statistically significant, in the case of *C. scutellarioides* “Electric Lime”, the greatest number of shoots were observed when NAA had been added, but not in the case of supplementation with cytokinins, which are known to abolish the apical dominance, thus causing plant branching. It is probable that there was a high amount of cytokinins in the initial material, and therefore, no significant effect after their supplementation was recorded.

In the case of plant in vitro culture, the application of appropriate physical factors, as well as hormones or their mixtures at a proper proportion, can induce a desirable morphological response of the plant. Investigations demonstrate that a combination of 0.5 mg dm^−3^ GA3 (gibberellic acid-3) and 0.5 mg dm^−3^ BAP (6-benzylaminopurine) caused a 1.4- and 1.3-fold increase, respectively, in the number of shoots and the plant height in comparison with those of the control. The application of 1.0 mg dm^−3^ IBA (indolylbutyric acid) prompted the earliest appearance of the roots, accompanied by a nearly 2.3-fold increase in the number of roots per stem as compared with the control medium [30]. Research also shows that breeding *C. scutellarioides* shoots on an MS medium beneath LED diodes combining red and green light is a good option for the stimulation of shoot proliferation in this plant [27].

The culture of callus and capillary roots can be applicable in a diversified multi-cascade production of secondary metabolites for industry. Biologically active compounds can be acquired without the usage of a whole plant. Production in a bioreactor is not limited by the biotic and abiotic factors involved with conventional cultivation. Callus was successfully induced on explants originating from stems through the application of a B5 (Gamborg medium) basic medium supplemented with 0.8 mg dm^−3^ 2,4-D(2,4-dichlorophenoxyacetic acid), 0.5 mg dm^−3^ NAA, or 2.0 mg dm^−3^ BA [28]. The team achieved callus production on a B5 medium enriched with 0.8 mg dm^−3^ 2,4-D after 15 days in darkness. Subsequently, in order to obtain a suspension culture, a callus of high looseness was separated, and transferred into a liquid medium, with an addition of 2.0 mg dm^−3^ BA, 0.5 mg dm^−3^ NAA, 0.8 mg dm^−3^ 2,4-D, and 600 mg dm^−3^ inositol [31]. The regeneration of callus was also achieved from young leaves cultured on a solidified MS medium supplemented with 1 mg dm^−3^ 2,4-D and 0.1 mg dm^−3^ kinetin. On explants from young leaves bred on an MS medium without phytohormones, tumor induction was carried out through transformation by *Agrobacterium tumefaciens* strains A281 and B6S3. Hairy roots were induced with *Agrobacterium rhizogenes* strain A4. Tumor and capillary roots were maintained on a hormone-free MS medium [6].

### 3.2. Discussion of Volatile Compounds

In plants, volatile bundles are formed on a few pathways: shikimate/phenylalanine, lipoxygenase (LOX), mevalonate (MVA), and 2-C-methyl-d-erythritol 4-phosphate (MEP) [32,33]. The influence of chemical compounds and phytohormones on the biosynthesis of volatile compounds in plants has previously been widely studied [34,35,36].

Plants are a rich source of chemical compounds used in various branches of industry, i.e., pharmaceutical or agricultural. Recently, research has emerged on the use of volatile compounds for appetite regulation [37]. The same appetite-inhibiting volatile compounds—limonene and nonanal—and appetite-reducing volatile compounds—camphene, β-pinene, sabinene, and p-cymene—were also identified in the specie studied here. 1-octen-3-ol and 3-octanone, at high doses, exhibit pesticidal properties against a range of invertebrate pests [38]. Commonly, they kill or repel juvenile entomopathogenic nematodes [39]. These substances show potential for use in future crop protection, as they exhibit a variety of interactions with invertebrates [40]. α-pinene exerts neuroprotective effects via anti-inflammatory and anti-apoptotic mechanisms [41]. Studies have shown that olfactory stimulation with α-pinene induces physiological relaxation [42]. In addition, it shows anticancer, antibacterial, antifungal, anti-leishmania, anti-inflammatory, antioxidative, neuroprotective, gastroprotective, antiapoptotic, antitumor, antimetastatic, insecticidal, and nematicidal properties [43,44,45]. α-selinene is found in large quantities in the composition of plant oils, exhibiting anticancer properties, as well as antioxidant, antifungal, and pharmacological activities [46,47,48,49,50]. Studies have shown that camphene regulated lipid metabolism; it can be identified for use in the treatment of lipid muscle atrophy, achieving controlled skeletal muscle atrophy, and also reducing plasma cholesterol and triglycerides in hyperlipidemic rats [51,52]. In addition, it exhibits anti-inflammatory, antidiabetic, hypolipidemic, anti-cancer, antiparasitic, antibacterial, antifungal, antioxidant, antiviral and anti- leishmania properties, plus some enzyme inhibitory activity [53].

Our studies showed the largest amounts for the volatile compounds of 1-octen-3-ol, 3-octanol, α-pinene, α-selinene, and campenein in *C. scutellarioides* “Electric Lime”. 1-octen-3-ol is used as a food additive. It is used as an attractant for insects such as tsetse flies, Anopheles, and Aedes mosquitoes [54,55]. Moreover, it reduces brown rot and the development of the plant fungal pathogen *Monilinia fructicola* (G. Winter) Honey, as well as induces some defense genes in plants [56,57]. 3-octanone can be sued as a lure and kill agent for the control of the brown garden snail [58].

Among the volatile compounds identified by another research team in the essential oil from an unknown variety of *C. scutellarioides*, the following occurred at the highest quantities: phytol, germacrene-D, caryophyllene, neophytadiene, Δ-cadinene, α-humulene, 6,10,14-trimethyl-2-pentadecanone, and copaene [14]. In the essential oil from leaves of another undefined genotype of *C. scutellarioides*, the following constituents showed a profuse representation: spathulenol, germacrene-D, bicyclogermacrene, nonacosan, and morillol [15]. During a GC-MS analysis of a *C. scutellarioides* methanol extract, the highest contents of furfural, 4H-pyran-4-on, 2,3-dihydro-3,5-dihydroxyl-6-methylo-, 5-hydroxymethylfurfural, phytol and phenol, and 2,2′-methylenebis [6-(1,1-dimethylethyl)-4-methyl- were identified, among others [59].

Over the years, studies have been published concerning the effect of phytohormones on changes in the intensity of biosynthesis of plant compounds. Thus, 6-benzylaminopurine (BA) was found to enhance the production of volatile organic compounds in the rose, Korean mint *Agastache rugosa* (Fisch. & C.A. Mey.) Kuntze, peppermint *Mentha piperita* L., and *Lantana camara* L. [60,61,62,63]. It also improves the production of secondary metabolites such as menthon, menthol, menthofuran, and pulegone in *Mentha piperita* [62]. Other research on peppermint confirmed its stimulating effect on menthon [64]. In the species Mexican oregano *Lippia origanoides* Kunth, BA stimulated the biosynthesis of myrcene and p-cymene and increased the overall monoterpenes content but caused a drop in the content of carvone [65]. Microplantlets of the Cassumunar ginger (*Zingiber montanum* (J. Koenig) Link ex A. Dietr.) cultivated on a medium enriched with BAP produced higher amounts of 2,4-D methyl ester, linoleic acid methyl ester, dodecane, and 3-tert-butylphenol [66]. Furthermore, BAP was reported to increase the contents of fatty and unsaturated acids in the niger *Guizotia abyssinica* (L.f.) Cass, whereas in *Malus sieversii* (Ledeb.) M.Roem., it was found to inhibit the biosynthesis of anthocyanins [67,68].

Fluridone has an inhibiting effect on the biosynthesis of carotenoids [69]. The lack of carotenoids negatively affects the formation of cell membranes and contributes to leaf whitening. This negative effect is irreversible, but the newly formed leaves of *Cyathea delgadii* Sternb., when transferred onto a substratum devoid of fluridone, became green and fully functional [69,70]. Fluridone inhibits the desaturation of phytoene and disrupts seed dormancy [71]. There are many studies on fluridone which indicate that it is an inhibitor of the biosynthesis of ABA [72,73,74]. Moreover, it has been observed to delay the accumulation of anthocyanins in the European blueberry *Vaccinium myrtillus* L. [74]. Alas, the number of studies that pertain to the impact of fluridone on the biosynthesis of plant compounds is negligible, and therefore, they are not sufficient to determine the specific mechanism of how this phytohormone affects the organic compound biosynthetic pathways. Nevertheless, taking into account the reports of its impact on carotenoids, phytoene, and ABA, one can draw a conclusion that fluridone influences the plastid methylerythritol phosphate (MEP) pathway, which is one of the biosynthetic pathways of terpenes.

The application of naphthaleneacetic acid (NAA) was found to trigger an increase in the production of volatile compounds and flower oil in species representing the genus *Rosa* L. [61,75]. Addtionally, NAA enhances the biosynthesis of carvone in *Croton floribundus* Spreng. [76]. In the case of the muskmelon *Cucumis melo* L., supplementation with NAA intensified the production of different carotenes, which are responsible for the vivid coloration of the fruit and its nutritive value. This phytohormone was recorded to stimulate the synthesis of such carotenes as beta-carotene, lycopene, and lutein [77]. It was also reported to improve the production of anthraquinone in the callus of the noni *Morinda citrifolia* Zenk [78].

Indole-3-acetic acid (IAA) improves the biosynthesis of volatile organic compounds in Korean mint *Agastache rugosa* [63]. It enhances the photosynthetic efficiency, and improves the contents of essential oils, phenol, and flavonoids in the holy basil *Ocimum tenuiflorum* L. [79]. Moreover, this phytohormone significantly increases the amounts of particular phenolic compounds in plant organs in apple mint *Mentha rotundifolia* L. [80]. In Mexican oregano *Lippia origanoides* Kunth, IAA increases the content of carvacrol [65], whereas in sweet wormwood *Artemisia annua* L., it improves the biosynthesis of artemisinin and increases the trichome density [81]. Furthermore, it improves the photosynthetic process and biosynthesis of polysaccharides in *Chlorococcum humicola*, a species of green algae [82], and increases the contents of carbohydrates, proteins, lipids, chlorophyll a and b, carotenoids, and metabolites in algae *Euglena* sp. [83].

Other compounds which act as plant growth hormones also affect the processes of biosynthesis of volatile compounds. Supplementation with gibberellins was recorded to induce quantitative changes in 1,8-cineole in the common sage *Salvia officinalis* L. [84]. Methyl jasmonate brought about changes in the amounts of linalool and β-ocymene in the upland cotton *Gossypium hirsutum* L. [85], whereas triacontanol was found responsible for differences in the contents of menthol, isomentone, L-mentone, and methyl acetate in the wild mint *Mentha arvensis* L. [86].

Similarly, the present research reveals the impact of plant growth regulators on the quantitative changes in specific volatile compounds and on the modification of the volatile compound profile in plants.

## 4. Materials and Methods

### 4.1. Micropropagation

The initial material was composed of overground parts of *C. scutellarioides* “Electric Lime”, coming from the Lower Silesia Agri-Food Wholesale Center (Pl. Dolnośląskie Centrum Hurtu Rolno-Spożywczego S.A.). For induction of in vitro cultures, fragments of stems with nodes were used. Prior to proper disinfection, the plant fragments were treated with a detergent solution and then with 70% ethanol. The disinfection proper was carried out with the use of a Javel solution (1 volume of Javel to 3 volumes of sterile water), with which the primary explants were treated for 10 min. Subsequently, the plant fragments were rinsed three times in sterile water for 5, 8, and 12 min. The thus prepared material was introduced into the MS medium (Murashige and Skoog) [87] basic medium, with an addition of 30 g dm^−3^ of saccharose, and solidified with agar of pH = 5.8 before autoclaving. The cultures were left in the breeding room (21 °C, photoperiod 16/8). The acquisition of material for the experiment was performed through the cloning of *C. scutellarioides* “Electric Lime” on the MS basic medium, without plant growth regulators. The plants were transferred to a fresh medium every fourth week. Next, the cultivation was conducted on the MS breeding medium, supplemented with 30 g dm^−3^ of saccharose, and solidified with agar at pH = 5.8, with five consecutive plant growth regulators at a dose of 0.5 g dm^−3^. These were BA (6-benzylaminopurine), 2iP (2-isopentyladenine), IAA (indole-3-acetic acid), NAA (naphthyl-1-acetic acid), and fluridone, and to the control, MS medium without phytohormones added, was applied. To each basic medium, 30 g dm^−3^ of saccharose and 8 g dm^−3^ of agar were added, whereas the pH was raised up to 5.8 before autoclaving. The experiment was set up in five replications. A single replication covered five nodal explants in a breeding vessel. After seven weeks, the plants were subjected to phenotypic analysis for the following traits: the length of roots (in cm), the length of the overground part (in cm), and the number of roots and shoots.

### 4.2. Chemical Compounds Analysis—Aroma Profiling

By comminuting the material with a scalpel, homogeneous samples of leaves and shoots from plants bred under varied in vitro conditions and from a plant cultivated in vivo (the source plant from which in vitro culture had been induced) were obtained. The analysis of volatile compounds was not carried out on plants from in vitro cultures enriched with 2iP due to the small amount of plant material obtained. Volatile compound profiling was performed using a gas chromatograph coupled with a mass spectroscope (single quadrupole mass spectrometer, gas chromatograph Shimadzu GC-MS QP 2020, Shimadzu, Kyoto, Japan). The solid-phase microextraction SPME-ARROW-type fiber, a mixture of divinylbenzene, carboxene WR and polydimethylsiloxane (DVB/C-WR/PDMS), was applied. The analysis was carried out in three replications. Each sample (~500 mg of fresh plant material, respectively) was placed in a head-space-type vial with a volume of 20 mL, with 10 μL of 2-undecanone (10,000 ppm) as the internal standard (IS). Before extraction, the samples were preincubated for 5 min at a temperature of 60 °C. The extraction of volatile compounds was carried out at 60 °C for 30 min. Desorption of the extracted volatile part was conducted in an injection device for 3 min. The injector temperature equaled 250 °C. As the carrier gas, helium was used at a flow of 1.0 mL min^−1^, with split 10. Separation was accomplished using a Zebron ZB-5 capillary column (30 m, 0.25 mm, 0.25 μm of stationary phase; Phenomenex, Torrance, CA, USA). The temperature protocol for the column was as follows: 50 °C, increase by 4.0 °C min^−1^ up to 130 °C, then 10 °C min^−1^ up to 240 °C. The MS analysis was performed with the use of scans from 40 to 300 *m*/*z* and with the application of electron ionization (EI) at 70 eV. The analysis was carried out in three replications.

Experimental retention indices of the identified compounds, calculated on the basis of the mixture of n-alkanes C6–C30 (Sigma-Aldrich, Saint Louis, MO, USA), were obtained. Identification of the compounds was performed through a comparison of the obtained retention indices with the literature retention indices for chemical compounds, and the mass spectra acquired were referred to the mass spectra available from the libraries (NIST 17 Mass Spectral and Retention Index Libraries (NIST17) and NIST WebBook) and the pertinent literature [88]. Semi-quantitative Polyrandom marking was carried out for each compound through normalization of the peak surface and calculations based on the amount of the internal standard added. For the semiquantative evaluation, standard curves were not employed for any of the compounds found in the samples. Concentrations of the internal standard were compared with the concentrations of other compounds, adopting the overall coefficient of equipment response equaling 1 for all the volatile compounds detected.

The percentage shares of the specific compounds were calculated from the surface of all the obtained peaks. The mass of the specific volatile compounds was expressed as related to the amount of the internal standard added to each sample under study.

### 4.3. Statistical Analysis

The results were expressed as a mean for each replication, and standard deviation values (±SD) were calculated (Supplementary Table S1). One-factor analysis of variance (ANOVA) was performed in order to verify the lack of significant effect of the phytohormones supplemented to the in vitro cultures on the morphological traits of the plants and on the chemical composition in *C. scutellarioides* “Electric Lime”. Under both in vitro and in vivo conditions, the hypothesis assumed a lack of influence of the applied breeding medium on the chemical composition of the plants. Significant differences were estimated at the level of 0.05 and 0.01. When the variance analysis yielded a significant result, the Tukey significant difference test (HSD) was carried out in order to compare the mean values [89]. The outcome was subjected to statistical analysis with the help of Statistica program version 13.3. A matrix of correlation coefficients was calculated using Statistica program version 13.3 to show trends in the amounts of volatile compounds observed due to the supplementation of plant growth regulators.

## Figures and Tables

**Table 1:** Impact of cultivation conditions on the number and quantity of volatile compounds per standard in *C. scutellarioides* “Electric Lime”.

Quantity of a Compound per Internal Standard [µg g^−1^]									
Time [min]	Compound	Literature Retention Index	Calculated Retention Index	Cultivation Conditions					
				In Vivo	Control In Vitro	BA [0.5 mg dm^−3^]	Fluridone [0.5 mg dm^−3^]	NAA [0.5 mg dm^−3^]	IAA [0.5 mg dm^−3^]
6.4	2-Hexenal	822	838	-	15.57 ± 2.39 ^nm^	2.51 ± 0.18 ^n^	5.01 ± 1.65 ^n^	17.31 ± 3.24 ^nm^	13.48 ± 4.66 ^nm^
6.4	3-Hexen-1-ol	838	839	46.78 ± 9.80 ^nml^	119.66 ± 12.17 ^nmlk^	41.40 ± 4.98 ^nml^	68.85 ± 13.08 ^nml^	144.78 ± 15.83 ^nmlkj^	92.11 ± 17.75 ^nmlk^
6.7	2-Hexen-1-ol	862	857	-	52.49 ± 17.68 ^nml^	4.12 ± 0.37 ^n^	12.39 ± 1.87 ^nm^	44.37 ± 7.67 ^nml^	23.84 ± 8.23 ^nml^
6.8	1-Hexanol	868	858	6.55 ± 1.09 ^n^	30.84 ± 4.59 ^nml^	17.36 ± 0.87 ^nm^	17.02 ± 3.75 ^nm^	39.50 ± 5.76 ^nml^	28.91 ± 4.29 ^nml^
8.9	α-Pinene	935	940	458.43 ± 31.24 ^nmlkjihg^	590.82 ± 156.90 ^nmlkjihg^	665.35 ± 99.54 ^mlkjihgf^	833.88 ± 214.03 ^hgf^	637.49 ± 156.23 ^nmlkjihgf^	329.15 ± 51.61 ^nmlkjih^
9.4	Camphene	953	955	3.28 ± 1.55 ^n^	13.70 ± 1.35 ^nm^	55.74 ± 45.13 ^nml^	38.26 ± 6.49 ^nml^	90.07 ± 33.07 ^nmlk^	135.59 ± 25.74 ^nmlk^
10.4	Sabinen	976	979	202.69 ± 37.45 ^nmlkjih^	121.62 ± 36.26 ^nmlk^	139.29 ± 40.54 ^nmlk^	367.97 ± 108.69 ^nmlkjih^	122.85 ± 35.01 ^nmlk^	115.71 ± 24.83 ^nmlk^
10.5	β-pinene	978	981	84.47 ± 6.91 ^nmlk^	95.96 ± 24.65 ^nmlk^	131.44 ± 33.74 ^nmlk^	145.78 ± 34.80 ^nmlkj^	123.57 ± 27.51 ^nmlk^	98.22 ± 15.12 ^nmlk^
10.6	1-Octen-3-ol	982	984	433.11 ± 41.43 ^nmlkjih^	3953.76 ± 486.22 ^b^	2772.06 ± 100.49 ^d^	3494.34 ± 497.67 ^cb^	4815.90 ± 882.53 ^a^	2882.59 ± 469.36 ^dc^
10.9	3-Octanone	989	990	23.31 ± 6.69 ^nml^	111.03 ± 8.66 ^nmlk^	41.88 ± 2.41 ^nml^	92.63 ± 15.94 ^nmlk^	149.26 ± 15.23 ^nmlkj^	153.91 ± 34.77 ^nmlkji^
11.0	β-Myrcene	992	994	10.46 ± 1.12 ^nm^	18.13 ± 3.63 ^nm^	21.14 ± 4.71 ^nml^	24.61 ± 5.26 ^nml^	22.92 ± 2.79 ^nml^	19.52 ± 2.40 ^nm^
11.2	3-Octanol	995	997	143.98 ± 16.14 ^nmlkj^	1279.46 ± 211.98 ^fe^	461.19 ± 11.92 ^nmlkjihg^	1100.13 ± 112.90 ^gfe^	1674.66 ± 355.52 ^e^	808.82 ± 85.25 ^ihgf^
11.5	α-Phellandrene	1002	1006	48.66 ± 10.94 ^nml^	42.75 ± 11.06 ^nml^	18.03 ± 6.67 ^nm^	41.98 ± 15.16 ^nml^	22.17 ± 5.61 ^nml^	16.79 ± 3.28 ^nm^
11.6	3-Hexen-1-ol acetate	1005	1010	35.75 ± 8.64 ^nml^	3.14 ± 0.83 ^n^	14.27 ± 12.09 ^nm^	1.45 ± 0.34 ^n^	9.62 ± 0.79 ^nm^	7.43 ± 1.72 ^nm^
12.2	p-Cymene	1026	1029	29.24 ± 2.58 ^nml^	29.74 ± 6.76 ^nml^	21.47 ± 3.16 ^nml^	26.91 ± 5.20 ^nml^	22.05 ± 4.92 ^nml^	11.18 ± 1.53 ^nm^
12.4	Limonene	1032	1033	63.04 ± 6.72 ^nml^	75.27 ± 16.79 ^nmlk^	79.79 ± 19.49 ^nmlk^	106.95 ± 23.13 ^nmlk^	79.98 ± 16.67 ^nmlk^	71.81 ± 13.03 ^nmlk^
12.5	Eucalyptol	1033	1036	0.96 ± 0.29 ^n^	2.71 ± 0.62 ^n^	5.13 ± 0.80 ^n^	3.08 ± 0.61 ^n^	23.33 ± 19.23 ^nml^	4.87 ± 0.67 ^n^
15.1	Linalool	1103	1101	16.11 ± 1.50 ^nm^	61.27 ± 8.18 ^nml^	38.83 ± 4.98 ^nml^	97.84 ± 17.56 ^nmlk^	79.44 ± 15.77 ^nmlk^	78.16 ± 13.12 ^nmlk^
15.3	Nonanal	1111	1106	15.18 ± 4.83 ^nm^	15.39 ± 5.05 ^nm^	6.31 ± 0.49 ^n^	10.58 ± 1.81 ^nm^	6.97 ± 1.37 ^n^	10.33 ± 3.92 ^nm^
21.9	Bornyl acetate	1284	1288	0.13 ± 0.06 ^n^	2.53 ± 1.46 ^n^	55.14 ± 53.56 ^nml^	7.61 ± 1.27 ^nm^	39.17 ± 27.01 ^nml^	158.20 ± 34.67 ^nmlkji^
24.4	Cyclosativene	1368	1376	7.94 ± 3.67 ^nm^	17.28 ± 3.91 ^nm^	16.26 ± 1.95 ^nm^	28.74 ± 5.31 ^nml^	18.55 ± 0.93 ^nm^	10.82 ± 2.03 ^nm^
24.7	Copaene	1382	1383	556.89 ± 64.50 ^nmlkjihg^	451.63 ± 65.62 ^nmlkjihg^	324.44 ± 18.68 ^nmlkjih^	439.41 ± 99.17 ^nmlkjih^	678.83 ± 112.12 ^lkjihgf^	474.52 ± 94.36 ^nmlkjihg^
24.8	Di-*epi*-α-cedrene	1385	1389	4.79 ± 1.12 ^n^	6.65 ± 2.21 ^n^	4.47 ± 0.91 ^n^	2.36 ± 0.56 ^n^	15.60 ± 6.20 ^nm^	21.72 ± 4.98 ^nml^
25.0	β-Elemene	1394	1396	13.67 ± 5.64 ^nm^	29.52 ± 8.71 ^nml^	17.19 ± 4.94 ^nm^	59.44 ± 12.79 ^nml^	90.47 ± 11.29 ^nmlk^	77.86 ± 16.80 ^nmlk^
25.5	Santalene	1417	1418	16.13 ± 6.12 ^nm^	30.84 ± 7.95 ^nml^	29.43 ± 3.23 ^nml^	63.34 ± 16.39 ^nml^	48.10 ± 10.71 ^nml^	28.79 ± 5.26 ^nml^
25.7	Caryophyllene	1424	1428	68.19 ± 13.08 ^nml^	74.04 ± 15.96 ^nmlk^	37.80 ± 2.96 ^nml^	51.54 ± 6.10 ^nml^	124.70 ± 25.14 ^nmlk^	172.60 ± 32.90 ^nmlkji^
26.2	δ-Guaiene	1452	1455	72.10 ± 32.80 ^nmlk^	131.98 ± 29.36 ^nmlk^	119.01 ± 12.91 ^nmlk^	257.29 ± 67.63 ^nmlkjih^	188.24 ± 53.83 ^nmlkjih^	159.90 ± 30.39 ^nmlkji^
26.8	γ-Selinene	1479	1484	10.33 ± 1.40 ^nm^	18.36 ± 0.92 ^nm^	14.02 ± 0.03 ^nm^	18.69 ± 4.29 ^nm^	40.06 ± 4.64 ^nml^	38.07 ± 9.48 ^nml^
26.9	β-Chamigrene	1776	1488	11.74 ± 2.59 ^nm^	16.19 ± 4.02 ^nm^	8.35 ± 1.14 ^nm^	128.86 ± 44.60 ^nmlk^	16.27 ± 5.29 ^nm^	74.88 ± 19.32 ^nmlk^
26.9	Germacrene D	1481	1491	48.55 ± 8.57 ^nml^	47.91 ± 3.91 ^nml^	17.27 ± 0.46 ^nm^	29.51 ± 15.62 ^nml^	79.81 ± 10.28 ^nmlk^	87.15 ± 13.81 ^nmlk^
27.2	α-Selinene	1494	1504	239.47 ± 49.92 ^nmlkjih^	478.64 ± 65.16 ^nmlkjihg^	291.59 ± 4.57 ^nmlkjih^	588.20 ± 142.43 ^nmlkjihg^	798.35 ± 82.56 ^jihgf^	728.95 ± 139.46 ^kjihgf^
27.2	α-Muurolene	1499	1508	8.41 ± 2.62 ^nm^	36.63 ± 16.23 ^nml^	19.02 ± 7.28 ^nm^	45.69 ± 17.47 ^nml^	45.02 ± 14.83 ^nml^	7.45 ± 1.46 ^nm^
27.5	γ i δ-Cadinene	1513	1525	168.25 ± 70.16 ^nmlk^	215.98 ± 81.53 ^nmlk^	118.72 ± 45.39 ^nml^	180.36 ± 70.28 ^nmlk^	269.68 ± 103.93 ^nmlkjih^	215.64 ± 87.24 ^nmlk^
		1524	1534						

Values after which there is another letter are significantly different (*p* > 0.05, according to the Tukey test).

**Table 2:** Impact of cultivation conditions on the percentage share of particular volatile compounds in *C. scutellarioides* “Electric Lime”.

Content of Volatile Compounds [%]									
Time [min]	Compound	Literature Retention Index	Calculated Retention Index	Cultivation Conditions					
				In Vivo	Control In Vitro	BA [0.5 mg dm^−3^]	Fluridon [0.5 mg dm^−3^]	NAA [0.5 mg dm^−3^]	IAA [0.5 mg dm^−3^]
6.4	2-Hexenal	822	838	-	0.19 ± 0.03 ^Δz^	0.04 ± 0.00 ^Δ^	0.06 ± 0.01 ^Δ^	0.16 ± 0.02 ^Δb^	0.18 ± 0.05 ^Δzd^
6.4	3-Hexen-1-ol	838	839	1.54 ± 0.23 ^Δzyxwvtsr^	1.44 ± 0.12 ^Δzyxwvtsr^	0.73 ± 0.11 ^Δzyx^	0.80 ± 0.05 ^Δzyxw^	1.36 ± 0.07 ^Δzyxwvts^	1.25 ± 0.17 ^Δzyxwv^
6.7	2-Hexen-1-ol	862	857	-	0.60 ± 0.14 ^Δzy^	0.07 ± 0.01 ^Δ^	0.15 ± 0.02 ^Δ^	0.42 ± 0.07 ^Δz^	0.31 ± 0.10 ^Δz^
6.8	1-Hexanol	868	858	0.21 ± 0.02 ^Δz^	0.37 ± 0.02 ^Δz^	0.30 ± 0.01 ^Δz^	0.19 ± 0.01 ^Δz^	0.37 ± 0.01 ^Δz^	0.39 ± 0.02 ^Δz^
8.9	α-Pinene	935	940	15.63 ± 2.16 ^fe^	6.87 ± 1.49 ^nmlkj^	11.50 ± 0.96 ^ihgf^	9.38 ± 1.14 ^lkjih^	5.76 ± 0.70 ^urqponmlk^	4.53 ± 0.47 ^zyxwvutsrqponm^
9.4	Camphene	953	955	0.11 ± 0.04 ^Δ^	0.17 ± 0.03 ^Δz^	0.88 ± 0.68 ^Δzyxw^	0.46 ± 0.07 ^Δzy^	0.86 ± 0.37 ^Δzyxw^	1.82 ± 0.06 ^Δzyxwvutsr^
10.4	Sabinen	976	979	6.83 ± 1.19 ^nmlkj^	1.43 ± 0.42 ^Δzyxwvtsr^	2.36 ± 0.51 ^Δzyxwvutsrqpo^	4.10 ± 0.83 ^Δzyxwvutsrqponm^	1.10 ± 0.18 ^Δzyxwv^	1.60 ± 0.29 ^Δzyxwvtsr^
10.5	β-Pinene	978	981	2.88 ± 0.40 ^Δzyxwvutsrqpon^	1.12 ± 0.24 ^Δzyxwv^	2.24 ± 0.41 ^Δzyxwvutsrqp^	1.65 ± 0.14 ^Δzyxwvtsr^	1.14 ± 0.22 ^Δzyxwv^	1.34 ± 0.03 ^Δzyxwvt^
10.6	1-Octen-3-ol	982	984	14.44 ± 0.54 ^gfe^	47.35 ± 2.22 ^ba^	48.67 ± 2.37 ^a^	41.55 ± 3.36 ^dc^	44.23 ± 0.79 ^c^	39.01 ± 0.92 ^d^
10.9	3-Octanone	989	990	0.75 ± 0.16 ^Δzyx^	1.37 ± 0.21 ^Δzyxwvts^	0.74 ± 0.06 ^Δzyx^	1.08 ± 0.02 ^Δzyxwv^	1.45 ± 0.29 ^Δzyxwvtsr^	2.05 ± 0.29 ^Δzyxwvutsrqp^
11.0	β-Myrcene	992	994	0.35 ± 0.01 ^Δz^	0.22 ± 0.04 ^Δz^	0.36 ± 0.06 ^Δz^	0.28 ± 0.01 ^Δz^	0.22 ± 0.03 ^Δz^	0.27 ± 0.01 ^Δz^
11.2	3-Octanol	995	997	4.79 ± 0.23 ^yxwvutsrqponm^	15.19 ± 1.05 ^gfe^	8.12 ± 0.56 ^mlkji^	13.32 ± 1.65 ^hgf^	15.24 ± 0.66 ^gfe^	11.17 ± 0.64 ^jihg^
11.5	α-Phellandrene	1002	1006	1.57 ± 0.22 ^Δzyxwvtsr^	0.49 ± 0.07 ^Δzy^	0.31 ± 0.10 ^Δz^	0.47 ± 0.14 ^Δzy^	0.20 ± 0.02 ^Δz^	0.23 ± 0.04 ^Δz^
11.6	3-Hexen-1-ol. acetate	1005	1010	1.17 ± 0.21 ^Δzyxwv^	0.04 ± 0.00 ^Δ^	0.27 ± 0.23 ^Δz^	0.02 ± 0.01 ^Δ^	0.09 ± 0.01 ^Δ^	0.10 ± 0.02 ^Δ^
12.2	p-Cymene	1026	1029	0.98 ± 0.05 ^Δzyxw^	0.35 ± 0.07 ^Δz^	0.38 ± 0.07 ^Δz^	0.31 ± 0.02 ^Δz^	0.20 ± 0.02 ^Δz^	0.15 ± 0.01 ^Δ^
12.4	Limonene	1032	1033	2.10 ± 0.10 ^Δzyxwvutsrqp^	0.88 ± 0.12 ^Δzyxw^	1.36 ± 0.24 ^Δzyxwvts^	1.23 ± 0.07 ^Δzyxwv^	0.74 ± 0.13 ^Δzyx^	0.97 ± 0.03 ^Δzyxw^
12.5	Eucalyptol	1033	1036	0.03 ± 0.01 ^Δ^	0.03 ± 0.01 ^Δ^	0.09 ± 0.01 ^Δ^	0.04 ± 0.00 ^Δ^	0.25 ± 0.21 ^Δz^	0.07 ± 0.01 ^Δ^
15.1	-Linalool	1103	1101	0.56 ± 0.13 ^Δzy^	0.73 ± 0.07 ^Δzyx^	0.67 ± 0.04 ^Δzy^	1.14 ± 0.02 ^Δzyxwv^	0.73 ± 0.03 ^Δzyx^	1.06 ± 0.02 ^Δzyxwv^
15.3	Nonanal	1111	1106	0.49 ± 0.12 ^Δzy^	0.18 ± 0.06 ^Δz^	0.11 ± 0.01 ^Δ^	0.13 ± 0.04 ^Δ^	0.06 ± 0.01 ^Δ^	0.17 ± 0.09 ^Δ^
21.9	Bornyl acetate	1284	1288	0.00 ± 0.00 ^Δ^	0.04 ± 0.02 ^Δ^	0.85 ± 0.82 ^Δzyxw^	0.09 ± 0.00 ^Δ^	0.43 ± 0.29 ^Δzy^	2.19 ± 0.44 ^Δzyxwvutsrqp^
24.4	Cyclosativene	1368	1376	0.25 ± 0.10 ^Δz^	0.20 ± 0.03 ^Δz^	0.29 ± 0.04 ^Δz^	0.33 ± 0.01 ^Δz^	0.18 ± 0.02 ^Δz^	0.15 ± 0.01 ^Δ^
24.7	Copaene	1382	1383	18.44 ± 0.27 ^e^	5.36 ± 0.18 ^vutsrqponml^	5.68 ± 0.22 ^utsrqponmlk^	5.04 ± 0.59 ^xwvutsrqponml^	6.27 ± 0.17 ^qponmlk^	6.40 ± 0.45 ^ponmlk^
24.8	Di-*epi*-α-cedrene	1385	1389	0.15 ± 0.02 ^Δ^	0.08 ± 0.02 ^Δ^	0.08 ± 0.01 ^Δ^	0.03 ± 0.00 ^Δ^	0.13 ± 0.03 ^Δ^	0.30 ± 0.05 ^Δz^
25.0	β-Elemene	1394	1396	0.48 ± 0.20 ^Δzy^	0.34 ± 0.08 ^Δz^	0.31 ± 0.10 ^Δz^	0.68 ± 0.06 ^Δzyx^	0.86 ± 0.14 ^Δzyxw^	1.03 ± 0.07 ^Δzyxwv^
25.5	Santalene	1417	1418	0.50 ± 0.17 ^Δzy^	0.36 ± 0.06 ^Δz^	0.51 ± 0.04 ^Δzy^	0.71 ± 0.10 ^Δzyx^	0.44 ± 0.05 ^Δzy^	0.39 ± 0.02 ^Δz^
25.7	Caryophyllene	1424	1428	2.22 ± 0.22 ^Δzyxwvutsrqp^	0.86 ± 0.07 ^Δzyxw^	0.66 ± 0.02 ^Δzy^	0.61 ± 0.04 ^Δzy^	1.14 ± 0.03 ^Δzyxwv^	2.33 ± 0.15 ^Δzyxwvutsrqpo^
26.2	δ-Guaiene	1452	1455	2.18 ± 0.95 ^Δzyxwvutsrqp^	1.54 ± 0.19 ^Δzyxwvtsr^	2.07 ± 0.13 ^Δzyxwvutsrqp^	2.89 ± 0.39 ^Δzyxwvutsrqpon^	1.67 ± 0.18 ^Δzyxwvtsr^	2.15 ± 0.08 ^Δzyxwvutsrqp^
26.8	γ-Selinene	1479	1484	0.34 ± 0.01 ^Δz^	0.22 ± 0.02 ^Δz^	0.25 ± 0.02 ^Δz^	0.21 ± 0.02 ^Δz^	0.38 ± 0.06 ^Δz^	0.50 ± 0.06 ^Δzy^
26.9	β-Chamigrene	1776	1488	0.38 ± 0.05 ^Δz^	0.19 ± 0.02 ^Δz^	0.14 ± 0.01 ^Δ^	1.41 ± 0.39 ^Δzyxwvtsr^	0.14 ± 0.02 ^Δ^	0.98 ± 0.11 ^Δzyxw^
26.9	Germacrene D	1481	1491	1.58 ± 0.13 ^Δzyxwvtsr^	0.58 ± 0.04 ^Δzy^	0.30 ± 0.02 ^Δz^	0.33 ± 0.16 ^Δz^	0.75 ± 0.09 ^Δzyx^	1.19 ± 0.06 ^Δzyxwv^
27.2	α-Selinene	1494	1504	7.76 ± 0.93 ^mlkji^	5.70 ± 0.03 ^usrqponmlk^	5.14 ± 0.37 ^wvutsrqponml^	6.67 ± 0.80 ^onmlk^	7.57 ± 0.88 ^mlkji^	9.78 ± 0.34 ^kjih^
27.2	α-Muurolene	1499	1508	0.27 ± 0.06 ^Δz^	0.40 ± 0.16 ^Δz^	0.34 ± 0.15 ^Δz^	0.49 ± 0.15 ^Δzy^	0.41 ± 0.11 ^Δz^	0.10 ± 0.01 ^Δ^
27.5	γ i δ-Cadinene	1513	1525	5.51 ± 2.19 ^uqponm^	2.55 ± 0.91 ^Δzyxwvutsrq^	2.08 ± 0.79 ^Δzyxwvtsr^	2.07 ± 0.74 ^Δzyxwvtsr^	2.51 ± 0.93 ^Δzyxwvutsrq^	2.93 ± 1.11 ^Δzyxwvutsrqpo^
		1524	1534						

Values after which there is a different letter are significantly different (*p* > 0.05, according to the Tukey test).

## Data Availability

Data is contained within the article or Supplementary Material or data is available on request.

## Supplementary Materials

The following supporting information can be downloaded at: https://www.mdpi.com/article/10.3390/molecules29102193/s1, Table S1. Matrix of correlation coefficients for the dependence of the synthesis of the analyzed volatile compounds on the cultivation conditions—the presence of applied phytohormones in relation to in vitro control conditions.
