# Supplementary material for: Plant In Vitro Cultures of Coleus scutellarioides (L.) Benth. “Electric Lime” and Possibilities of Modification in the Biosynthesis of Volatile Compounds
Source: Molecules. 2024 May 8;29(10):2193. doi: 10.3390/molecules29102193 (PMC11124424; doi:10.3390/molecules29102193)
Supplement: Supplementary file 1 [file molecules-29-02193-s001.zip › molecules-2946743-supplementary.pdf]

Table S1. Matrix of correlation coefficients for the dependence of the synthesis of the analyzed volatile compounds on the cultivation conditions - the presence of applied phytohormones in relation to *in vitro* control conditions.

Condition of cultivation	Range of volatile compounds 4815.9 – 144.0 μg g^{-1}	Range of volatile compounds 368.0 – 0.1 μg g^{-1}	Range of volatile compounds 87.2 – 0.0 μg g^{-1}
	Control *in vitro*	Control *in vitro*	Control *in vitro*
BA	0.98*	0.90*	0.82*
Fluridone	1.00*	0.79*	0.88*
NAA	1.00*	0.98*	0.82*
IAA	0.98*	0.88*	0.71*

*Correlations denoted by the correlation coefficient are significant with $p < 0.5000$.
